# Methamphetamine abuse impairs motor cortical plasticity and function

**DOI:** 10.1038/mp.2017.143

**Published:** 2017-07-25

**Authors:** X Huang, Y-Y Chen, Y Shen, X Cao, A Li, Q Liu, Z Li, L-B Zhang, W Dai, T Tan, O Arias-Carrion, Y-X Xue, H Su, T-F Yuan

**Affiliations:** 1 Shanghai Key Laboratory of Psychotic Disorders, Shanghai Mental Health Center, Shanghai Jiao Tong University School of Medicine, Shanghai, China; 2 School of Psychology, Nanjing Normal University, Nanjing, China; 3 Co-innovation Center of Neuroregeneration, Nantong University, Nantong, Jiangsu, China; 4 National Institute on Drug Dependence and Beijing Key Laboratory of Drug Dependence, Peking University, Beijing, China; 5 Rehabilitation Medicine Center, First Affiliated Hospital of Nanjing Medical University, Nanjing, China; 6 Da Lian Shan Institute of Addiction Rehabilitation, Nanjing, China; 7 Cangzhou Medical College, Cangzhou, Hebei, China; 8 Sichuan Provincial Hospital for Women and Children, Chengdu, China; 9 Unidad de Trastornos del Movimiento y Sueno, Hospital General Dr Manuel Gea Gonzalez, Mexico City, Mexico; 10 Key Laboratory for Neuroscience of Ministry of Education and Neuroscience, National Health and Family Planning Commission, Peking University, Beijing, China; 11 State Key Laboratory of Quality Research in Chinese Medicine, Institute of Chinese Medical Sciences, University of Macau, Taipa, Macau, China; 12 State Key Laboratory of Brain and Cognitive Sciences, The University of Hong Kong, Hong Kong

## Abstract

Exposure to addictive drugs triggers synaptic plasticity in reward-related brain regions, such as the midbrain, nucleus accumbens and the prefrontal cortex. Effects of chronic drug exposure on other brain areas have not been fully investigated. Here, we characterize synaptic plasticity in motor cortex after methamphetamine self-administration in rats. We show that this causes a loss of corticostriatal plasticity in rat brain slices and impaired motor learning in the rotarod task. These findings are paralleled by the observation of a lack of transcranial magnetic stimulation-induced potentiation or depression of motor evoked potentials in human patients with addiction, along with poor performance in rotary pursuit task. Taken together, our results suggest that chronic methamphetamine use can affect behavioral performance via drug-evoked synaptic plasticity occluding physiological motor learning.

## Introduction

Drug-evoked synaptic plasticity has been proposed as a neural substrate of adaptive behavior.^1, 2^ Initial exposure to an addictive drug leads to insertion of calcium-permeable AMPA (α-amino-3-hydroxy-5-methyl-4-isoxazolepropionic acid) receptors and calcium-impermeable *N*-methyl-D-aspartate (NMDA) receptors in dopamine (DA) neurons of the ventral tegmental area;^3, 4^ chronic drug exposure also alters synaptic transmission in medium spiny neurons of the nucleus accumbens synapses and, in some cases, also at cortical synapses.^5, 6^

Most of these studies focused on drug-evoked synaptic plasticity in subcortical regions (for example, ventral striatum, amygdala, ventral tegmental area) and prefrontal cortex,^7, 8^ all of which receive projections from midbrain DA neurons. Sensory and motor areas receive only sparse DA inputs and have not been extensively studied.^9^ However, evidence for drug-adaptive function also exists for these cortical regions. For instance, brain imaging studies in humans with cocaine or nicotine reveal a cue-induced activation of the sensory association cortex and motor cortex that reflects craving status and predicts relapse of the addicted patients.^10, 11, 12, 13^ Chronic exposure to methamphetamine causes structural deficits and altered DA receptor function in the cortical-striatal projections in humans.^14^ Animal studies further reported a cocaine-altered expression in genes of the dorsal striatum associated with motor learning.^15^ Finally, drug abuse accompanies compulsive behavior and drug seeking that could result from altered motor-striatal plasticity and functioning.

In the present study we characterized synaptic plasticity at the motor cortex to the dorsal striatum in rats self-administrated methamphetamine. We then combined transcranial magnetic stimulation (TMS) to monitor plasticity of the motor evoked potential (MEP) in humans to probe a potential plasticity deficit in methamphetamine abusers.

## Materials and methods

### Animal subjects

A total of 23 male Sprague–Dawley rats (260–300 g, Beijing University Animal Center, Beijing, China) were subjected for methamphetamine self-administration (SA) training as previously described^16, 17^ and 16 male rats were employed as the control (random assignment, animal sample size not preestimated). All animal experimental procedures have been approved by the ethic committee of animal research at Nanjing Normal University, Shanghai Jiaotong University and Beijing University.

For self-administration, the training chambers (AniLab Software & Instruments, Ningbo, Zhejiang, China) were equipped with two nose-poke detectors (AniLab Software & Instruments) located 5 cm above the floor of the chambers. Nose pokes in one (active) opening led to methamphetamine infusions that were accompanied by a 5 s tone–light cue. Nose pokes in the other (inactive) were also recorded but had no consequence. In the present study, all animals were included for analyses (not in blind manner).

### Human subjects

A total of 56 male addicts (methamphetamine only (METH) group, treatment seeking) (26–55 years old, right handed) were recruited for the present study and 35 healthy male subjects as controls (24–54 years old, right handed). There were no differences in age, smoking status or alcohol intake history between the two groups. All subjects reported no use of drugs in the 2 weeks before the investigation (abstinence from drug use ranged from 2 weeks to 3 months) and this was verified by urine sample examination; all subjects reported intact sleep before the experiment.

All the subjects recruited for the study participated voluntarily, and signed the written informed consent forms. Subjects reported no prior history of head trauma, epilepsy, heart diseases or metal implants in the body. The study has been approved by the ethics committee of Shanghai Mental Health Center and Nanjing Normal University, and all experimental procedures followed the guidelines of human medical research (Declaration of Helsinki). The clinical trial registration was ChiCTR-IOR-16008060 (Cortical plasticity of addicts) at http://www.chictr.org.cn.

### Criteria for METH abuse

The abuse of METH was defined by the following criteria: (1) only METH use (not used with other drugs, for example, heroin) for at least 2 years; (2) METH intake of minimum 3 times per week, for a period at least a month (many METH users change their intake frequency and those with occasional drug use were not included); (3) dosage of >0.3 g per day (range 0.3–2 g); and (4) did not receive additional drug therapy to quit addiction.

### Brain slice electrophysiology

For brain slice preparation, rats were deeply anesthetized with isoflurane and decapitated. Brains were quickly removed and submerged in cutting solution (in mM as follows: 119 NaCl, 2.5 KCl, 6 kynurenic acid, 1 NaH_2_PO_4_, 13.5 glucose, 100.1 sucrose, 77.9 NaHCO_3_, 3.5 CaCl_2_ and 7.3 MgCl_2_). Coronal sections (250 μm) containing motor cortex, dorsal lateral striatum and dorsal medial striatum were cut using a vibratome (VT1200S; Leica Microsystems, Wetzlar, Germany) in ice-cold cutting solutions. Brain slices were submerged in artificial cerebrospinal fluid (ACSF) (in mM as follows: 119 NaCl; 2.5 KCl, 1 NaH_2_PO_4_, 11 glucose, 26.2 NaHCO_3_, 2.5 CaCl_2_ and 1.3 MgCl_2_) at 27 °C for 30 min, and equilibrated with 95% O_2_ and 5% CO_2_. Slices were then individually transferred to the recording chamber and superfused continuously with 32 °C ACSF containing 50 μM picrotoxin.

For the recording of field population spikes, electrodes (3–5 MΩ) were filled with ACSF, and a stimulation intensity of 30% maximum response is employed. For whole-cell patch-clamp recording, the internal solution contained (in mM): 135 KMeSO_4_, 10 KCl, 10 HEPES, 10 Na2-phosphocreatine, 4 MgATP, and 0.3 Na_3_GTP (pH 7.2–7.4); or CsMeSO_4_ 130, NaCl 10, EGTA 10, MgATP 4, Na_3_GTP 0.3 and HEPES 10 (pH 7.2–7.4). For AMPA/NMDA ratio calculation, the AMPA receptor response was recorded at −70 mV to prevent NMDA receptor activation, and the NMDA receptor response was estimated at 50 ms after the peak of mixed currents at +40 mV.

All recordings were filtered (4 kHz low-pass filter) and sampled (10 kHz) for online and later offline analysis with Multiclamp 700B amplifier (Molecular Devices, Sunnyvale, CA, USA) and Clampfit 10.5 Software.

### Human TMS procedures

Intermittent TMS or continuous theta burst stimulation (cTBS) stimulation were performed as described in previous studies.^18, 19^ Briefly, high-frequency intermittent TMS (10 Hz, strength at 100% resting motor threshold, 5 s on and 10 s off for 10 min; 2000 pulses) or cTBS (50 Hz of 80% MT for 3 pulses train was repeated at 200 ms for 40 s, 600 pulses) were applied over the left primary motor cortex with a CCY-I TMS instrument (Yiruide, Wuhan, China) and an ‘8’-shaped coil for accurately targeted stimulation.

### Human MEP procedures

The MEPs were recorded from abductor pollicis brevis muscle on right hand using the CCY-I TMS affiliated MEP system and analyzed with the CCY-I TMS software. For MEP recording, 20 consecutive MEPs (5 s interval) at each time point were evoked by single pulse TMS stimulation at M1 region, and the peak values were averaged for each time point. Two points of baseline values were taken (separated by 5 min) and the plasticity protocol will be applied if there were no more than 10% differences between the two baseline values. After the plasticity protocol, MEPs were measured after 5, 10, 15 and 30 min, respectively.^20^

### Rotary rod task for rat

A rotary rod instrument (Shanghai Xinruan, Shanghai, China) was used in present study. Before the formal test, the animals were briefly familiarized with the rotating rod. The rotating speed was set for each animal to reach an initial latency to fall at ∼20 s. Then, the animals were tested for four times with a 10 min interval between tests.

### Rotary pursuit task for human subjects

In rotary pursuit motor learning task, the human subjects were asked to trace a point on the rotating plate with a probe (instrument from Bei Da Qing Niao, Beijing, China), and the time for the probe to successfully contact the rotating point was measured in 4 trials separated by 10 min each.^21^ Before the test, the subjects were familiarized with the test but were allowed less than three trials to prevent overlearning. The speed of the rotating plate was adjusted to reach a starting baseline between 10 and 25 s.

### Western blot assays of synaptosomal proteins

The crude synaptosomal membrane fraction from the motor cortex and dorsal striatum region was purified as described previously.^22^ Briefly, brains were removed acutely, and the regions of interests were homogenized by sonication in ice-cold homogenization buffer (0.32 M sucrose, 1 mM EGTA, 1 mM EDTA, 4 mM HEPES, pH 7.4) and a protease inhibitor cocktail at 1000 *g* at 4 °C for 10 min for pellet (P1, containing nuclei and large debris) preparation. Then the supernatant S1 was centrifuged at 10 000 *g* at 4 °C for 30 min for synaptosomal fraction (P2) and supernatant (S2). The crude synaptosomal membrane pellet (P2) was lysed hypo-osmotically and centrifuged at 25 000 *g* for at 4 °C for 30 min to generate the synaptosomal membrane fraction (LP1) and supernatant (LS1). Then, the synaptosomal membrane fraction was solubilized and denatured in Tris-buffered saline containing 0.2% SDS (70 °C, 10 min) diluted in 1% Triton X-100 and 5% glycerol and maintained in protease inhibitor cocktail (50 μM MG132 and 10 μM β-lac).

The 4 × loading buffer (16% glycerol, 20% mercaptoethanol, 2% SDS and 0.05% bromophenol blue) was added to each sample (3:1 sample/loading buffer) before boiling for 3 min. After coolling down, the samples were subjected to SDS–polyacrylamide gel electrophoresis (10% acrylamide/0.27% *N*,*N*’-methylenebisacryalamide resolving gel) for ~40 min at 80 V in stacking gel and ~1 h at 130 V in resolving gel. The proteins were electrophoretically transferred onto Immobilon-P transfer membranes (Millipore, Bedford, MA, USA) at 0.25 A for 2.5 h.

Primary antibodies included anti-GluA1 antibody (1:1000; Abcam, Cambridge, MA, USA, ab109450), anti-GluA2 antibody (1:1000; Abcam; ab52932), anti-GluA3 antibody (1:1000; Abcam, ab40845), anti-GluN2A antibody (1:1000; CST, 4205), anti-GluN2B antibody (1:1000; CST, 4207), anti-GluN3A antibody (1:1000; Santa Cruz, Dallas, TX, USA, sc-51160) or anti-Na-K ATPase antibody (1:1000; Abcam, ab7671). Following overnight incubation at 4 °C, the membranes were incubated for 45 min at room temperature on a shaker with horseradish peroxidase-conjugated secondary antibody (goat anti-rabbit IgG, ZSGB-BIO, ZB-2301; goat anti-mouse IgG, ZSGB-BIO, ZB-2305) diluted 1:5000 in blocking buffer. The blots were then screened using the chemiDoc MP system (Bio-Rad, Hercules, CA, USA) for 5–60 s. The level of protein expression was normalized to Na-K ATPase and analyzed with Quantity One software (version 4.4.0; Bio-Rad).

### Statistics

Mean and s.e.m. represented data. Intergroup differences were compared by using independent sample *t*-test with Origin software (Northampton, MA, USA). *P*<0.05 was considered as statistically significant.

## Results

### Impaired cortical-striatal plasticity and motor learning after METH SA in rats

**Figure 1:**
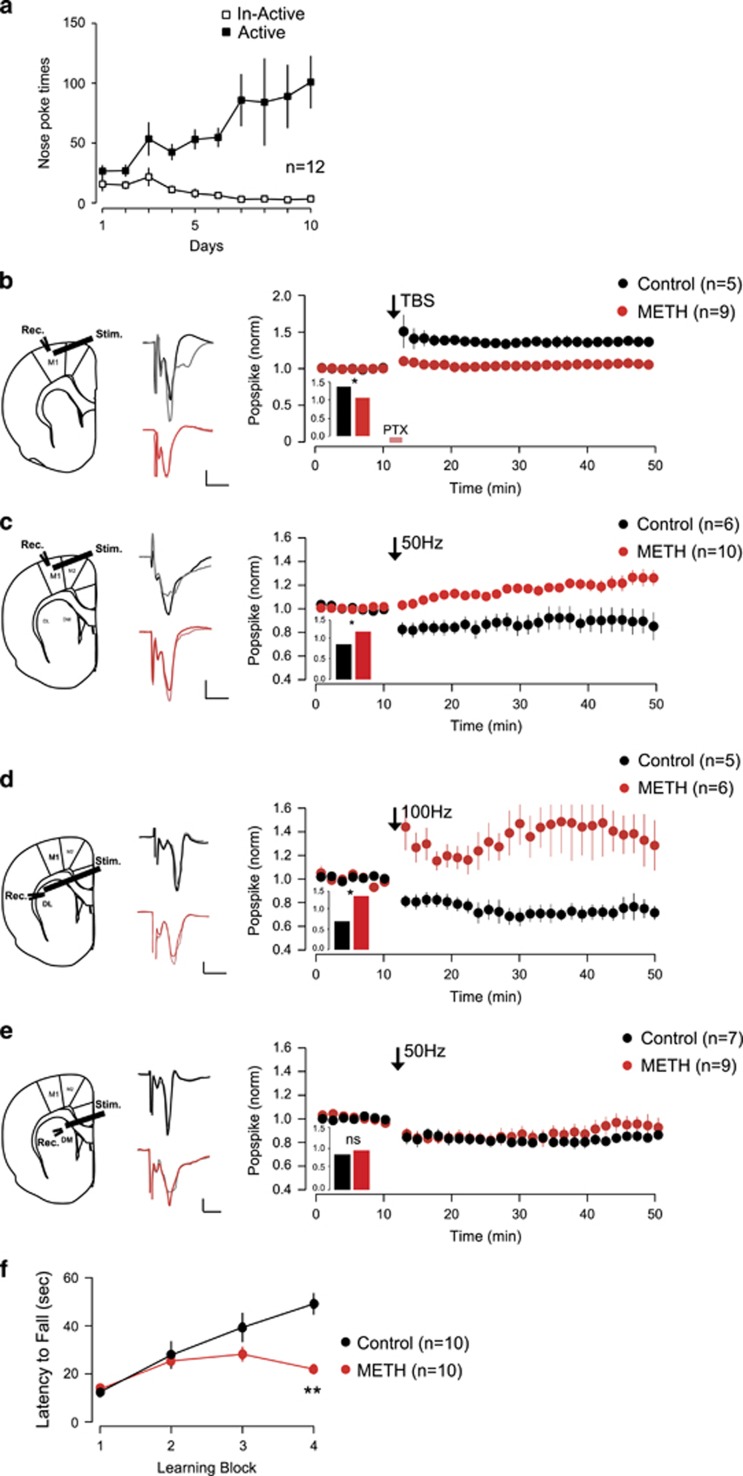
Impaired cortical-striatal plasticity and motor skill learning in self-administrated (SA) rats. (**a**) Nose-poke data of methamphetamine (METH) SA rats (*n*=12). (**b**) Motor cortical–cortical long-term potentiation (LTP)-like plasticity was impaired in SA rats (*n*=5 for control, *n*=9 for METH, *P*<0.05). (**c**) Motor cortical–cortical long-term depression (LTD)-like plasticity was impaired in SA rats (*n*=6 for control, *n*=10 for METH, *P*<0.05). (**d**) Motor-dorsal lateral striatum plasticity was impaired in SA rats (*n*=5 for control, *n*=6 for METH, *P*<0.05). (**e**) Motor-dorsal medial striatum plasticity remains intact in SA rats (*n*=7 for the control group, *n*=9 for METH, *P*>0.05). Scale bar for all field excitatory postsynaptic potential (fEPSP) example traces in (**c–f**): 0.2 mV, 50 ms. From left to right: recoding site, example trace (gray for after the protocol), group data. Black filled circle: control group; red filled circle: METH group. (**f**) Motor skill learning was impaired in METH SA rats. Motor skill learning curve was constructed by measuring the latency to fall from the rotating rod (*n*=10; METH vs control, *P*<0.01, *P<0.05 and **P<0.01).

Rats were trained to SA METH for 10 days (Figure 1a).

After 3–9 days of withdrawal, we recorded field potentials in slices of the motor cortex (M1) and the dorsal medial striatum evoked by electrically stimulation as described in a previous study.^23^ We used TBS combined with transient GABA_A_ receptor blockade to induce long-term potentiation and 50 Hz stimulation to elicit long-term depression in motor cortex;^24^ we used 100 and 50 Hz stimulation to induce long-term depression in dorsolateral (DL) striatum and dorsomedial striatum,^23^ respectively. We found that these stimlation protocols failed to induce synaptic plasticity in METH SA group (Figures 1b–d) in both the motor cortex and DL striatum, whereas there was no difference in the dorsomedial striatum. This points to a METH effect in the DL, the preferential target of M1 in habit-related drug seeking.^25^ Given the established role of DL striatum in motor learning, we tested the rats for motor skills on a rotarod task. We found that the learning curve of animals in the METH SA group plateaued after the second block (Figure 1f).

### Altered synaptic transmission in cortical-striatal synapses

**Figure 2:**
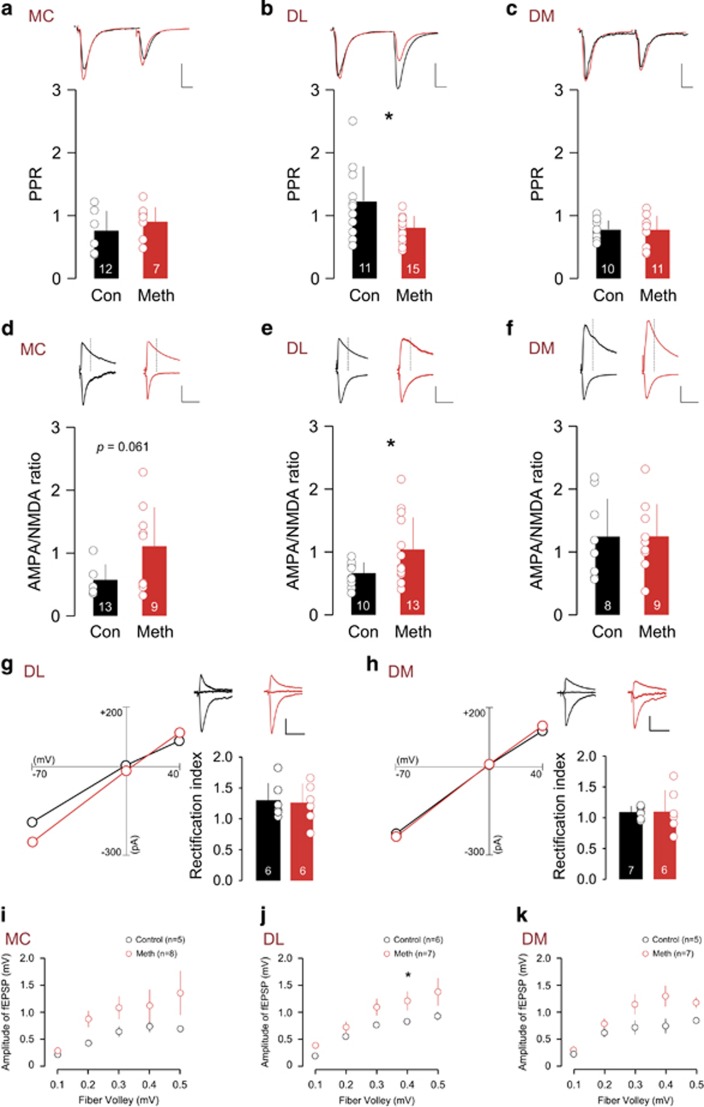
Altered functioning in cortical-striatal synapses. Synaptic transmission parameters (paired-pulse ratio (PPR) (**a–c**), AMPA/NMDA (A/N) ratio (**d–f**), retification index (RI) (**g–h**), input -output curve (**i–k**)) for Motor cortex (MC), dorsolateral (DL) and dorsomedial (DM) pathway synapses. *P<0.05. There were no significant changes in DM and MC synapses, whereas DL pathway revealed decreased PPR value (enhanced presynaptic release) and increased A/N ratio (enhanced postsynaptic functions). Scale bars for (**a–c**): 100 pA, 10 ms. Scale bars for (**d–h**): 100 pA, 50 ms. Dashed line in (**d–f**) indicates the estimated NMDA receptor (NMDAR) peak at +40 mV for AMPA/NMDA ratio calculation. AMPA, α-amino-3-hydroxy-5-methyl-4-isoxazolepropionic acid; MC, motor cortex; NMDA, *N*-methyl-D-aspartate; RI, rectification index.

To investigate the expression mechanims of METH evoked synaptic plasticity, we established the input–output function, AMPA/NMDA ratio, and the paired-pulse ratio. We found no change in these parameters in the motor cortex M1 (Figures 2a, d and i). In the striatum, neurons of the DL (Figures 2b) but not of the dorsomedial striatum (Figures 2c, f and k) showed a decreased paired-pulse ratio, an increased AMPA/NMDA ratio and steeper input–output function, reflecting enhanced synaptic transmission via increased presynaptic release and upregulated postsynaptic sensitivity.

**Figure 3:**
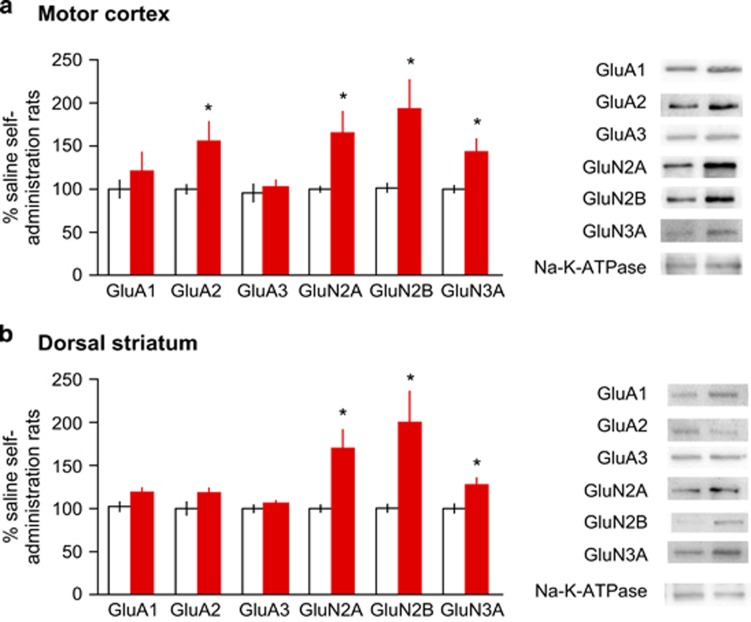
Molecular changes of synaptic glutamate receptors at cortical-striatal synapses. (**a**) Effects of methamphetamine self-administration (METH SA) on synaptic proteins in the motor cortex. (**b**) Effects of METH SA on synaptic proteins in dorsal striatum. *P<0.05 (*n*= 6; METH vs control).

To examine the subunit composition of AMPA and NMDA receptors, we performed western blot analyses of synaptosomal proteins on both motor cortex and dorsal striatum. Interestingly, we detected increased amount of GluN2A/2B/3A expression in both the motor cortex and the dorsal striatum, suggesting the insertion of the heterotrimeric GluN3A-containing (calcium-impermeable) NMDA receptors (Figures 3a and b). On the other hand, GluA2 protein levels were unaltered, in line with the linear I–V curve after drug exposure, indicating the presence of GluA2-containing AMPARs (Figures 2g and h).

### Diminished motor cortical plasticity and impaired motor learning in human addicts

**Figure 4:**
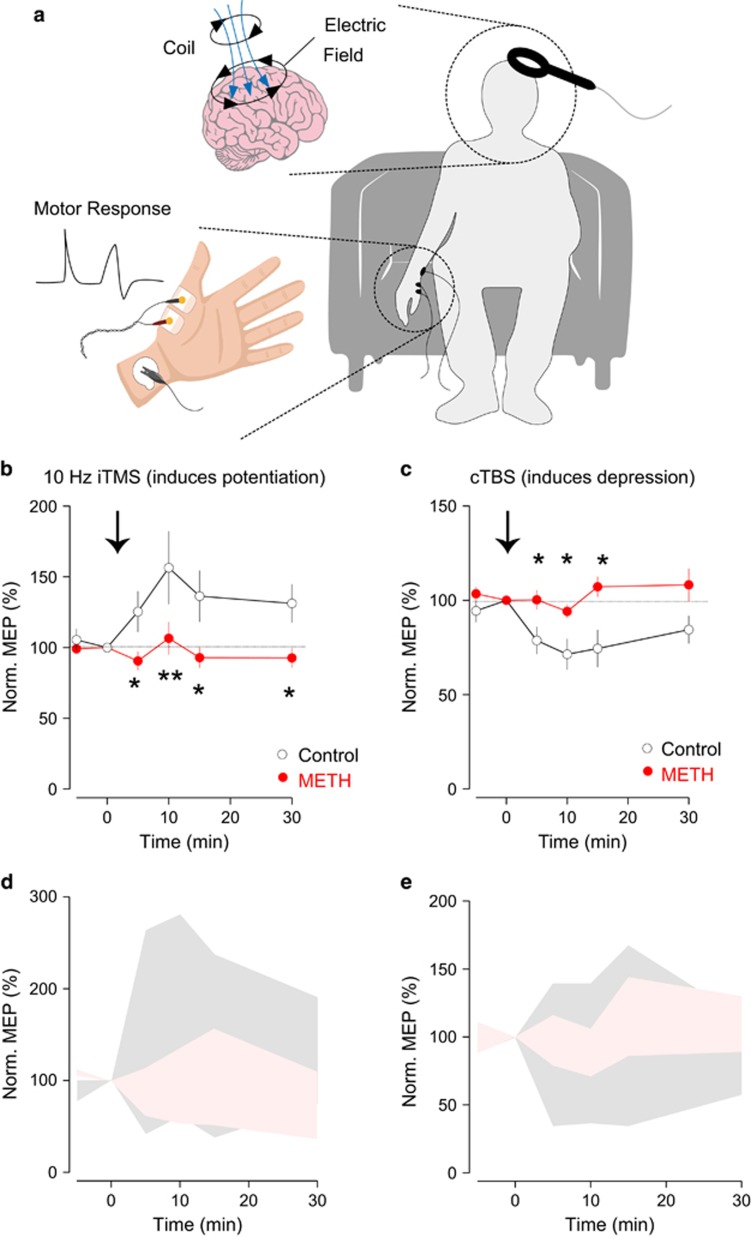
Diminished cortical plasticity in addicts. (**a**) The design of transcranial magnetic stimulation (TMS) and motor evoked potential (MEP) recordings. Single TMS pulse was administrated on the left motor cortex, and the MEP signal was recorded from abductor pollicis brevis (APB) muscle on right hand. Modified from Shen *et al.*^20^ with permission. (**b**) The 10 Hz repetitive TMS (rTMS) stimulation protocol results in potentiation of MEPs in control but not methamphetamine (METH) group (*n*=11 for control and *n*=18 for METH); (**c**) continuous theta burst stimulation (cTBS) protocol results in depression of MEPS in control but not METH group (*n*=9 for control and *n*=11 for METH); (**d**, **e**) control group MEPs exhibit more variance after 10 Hz cTBS protocol. Black open circle: control group; red filled circle: METH group. *P<0.05.

Next, we examined the impact of chronic METH use on motor cortical plasticity and motor skill learning in human addicts. We used distinct TMS protocols to potentiate (10 Hz, 10 min) or depress (cTBS, 48 s) MEPs (Figure 4a). In the control group, 10 Hz repetitive TMS stimulation aimed at the motor cortex potentiated MEPs for at least 30 min. In constrast, this potentiation was not obsereved in the group of METH addicts (METH group) (Figure 4b). Similarly, cTBS protocol failed to induce a depression of MEPs in METH group that was robustly observed in the control group (Figure 4c). Notably, with both stimulation protocols, the control group exhibited more individual variability compared with the METH group (Figures 4d and e). Last but not least, the craving score in the METH addict, measured by subjective responses to a craving scale, inversely correlated with the MEP plasticity at 10 min time point (Pearson’s *r*=−0.6315; adjusted *r*^2^=0.30).

**Figure 5:**
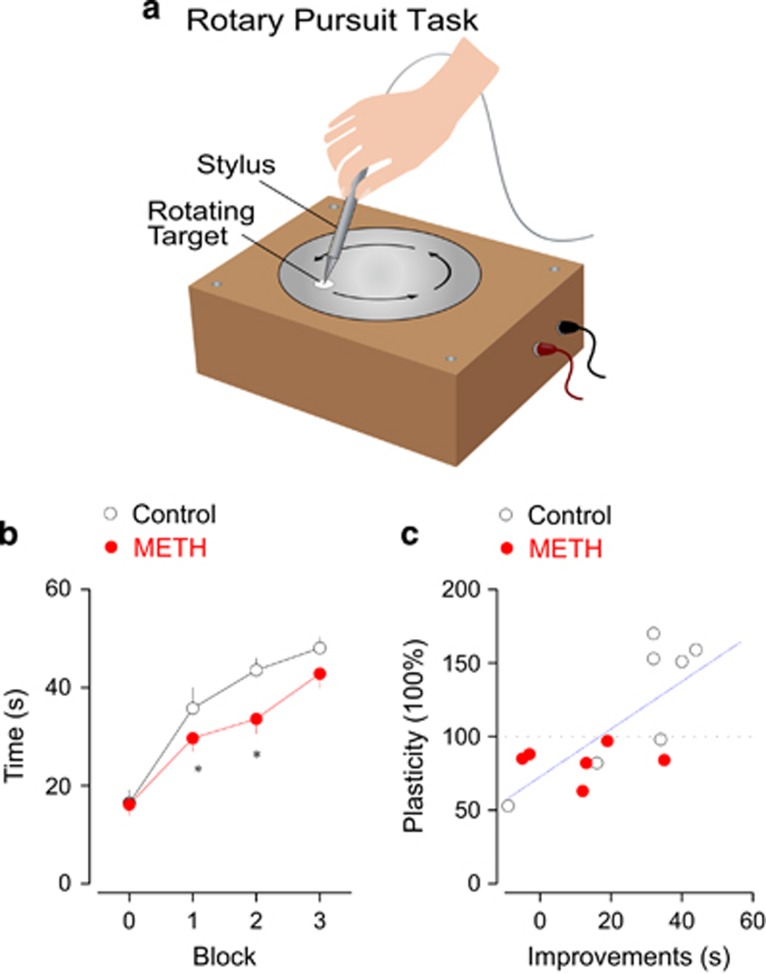
Impaired motor learning correlates with the decreased plasticity. (**a**) In rotary pursuit motor learning task, the subjects were asked to trace a point on the rotating plate with a probe. Each block lasts for 60 s (interval at 10 min) and the success of time with probe targeting the point was recorded. Modified from Zhou *et al.*^21^ with permission. (**b**) The methamphetamine (METH) group exhibits worse performance during rotary pursuit learning task (*n*=7 for control and *n*=15 for METH), *P*<0.05 for second and third tests. (**c**) The motor learning performance at block two was correlated to the plasticity induction changes at the same time point (Pearson’s *r*=0.705, *P*=0.004). Black open circle: control group; red filled circle: METH group.

Finally, we tested the motor skill learning ability in addicts with a rotary pursuit learning task (Figure 5a). The subjects in the METH group took more time to learn the task compared with the control group (Figure 5b). Notably, the motor learning performance correlated with the plasticity of the MEP elicited by 10 Hz stimulation protocol (Figure 5c).

## Discussion

We report that METH abuse can affect plasticity of the motor cortex in both drug-exposed rats and human addicts. The reduced cortical plasticity in addicts argues for an involvement of motor system in advanced stages of the disease, in line with the hypothesis that with the progression of addiction, the dorsal striatum becomes involved.^8, 25, 26^ Moreover, the poor learning ability could reflect a reduced flexibility that contributes to maintenance of the compulsive behavior and inhibits recovery.

One potential molecular correlate of a reduced synaptic plasticity is the insertion of calcium-impermeable (GluN3A-containing) NMDA receptors that were detected on midbrain DA neuron at an early stage of drug exposure,^3^ as well as in striatal neurons in degenerative diseases.^27^ NMDA receptors in the cortex are also essential for many forms of plasticity, and the presence of calcium-impermeable NMDA receptors alter the rules for plasticity induction.^28^ Here we confirm the insertion of GluN3A by western blot, supporting the notion that a failed induction mechanism underlies the impaired bidirectional plasticity.

Another mechanism underlying the diminished cortical plasticity may be the loss of dopaminergic signaling.^29^ For instance, human METH addicts exhibit decreased DA transmission in the brain that may manifest as drug-induced parkinsonism.^30, 31, 32, 33^ In the present study we found a delayed motor learning ability in addicted patients. Future studies will be required to understand whether other types of motor learning than the pursuit task tested here are also impaired after drug abuse.

We also provide evidence for a decreased intrinsic excitability of neurons in cortical-striatal circuits after repeated METH exposure.^2, 34^ The reduced response to TMS protocols could be partly explained by the failure to reach the firing threshold in neurons that are less excitable.

One limitation of the present study is that with electrical stimulation, the exact synaptic pathways in the motor-striatal circuit cannot be reliably identified. Employing optogenetic tools that allow further dissection will be helpful to identify the circuits affected by these changes. On the human study side, the TMS-induced cortical plasticity can be explained by either synaptic plasticity or changes of intrinsic excitability. To refine stimulation protocols that could restore normal plasticity, it will be important to examine which of the two potential mechanisms applies.

In summary, the present study demonstrates an unexpected change in motor cortical plasticity after METH abuse. Clinical studies are needed to explore the relationships between altered motor cortical plasticity and addiction symptoms (for example, craving, relapse). This will enable translation to the clinic of rational therapy for drug addiction, a disease with a tremendous burden for society.
